# Comparison of intracompartment pressure changes in tibial plateau fractures and controlled people: A pilot study

**DOI:** 10.1371/journal.pone.0312526

**Published:** 2024-10-29

**Authors:** Jialiang Guo, Jianfeng Zhang, Kezheng Du, Weichong Dong, Xiaohui Han, Yingze Zhang, Zhiyong Hou

**Affiliations:** 1 Department of Orthopaedics, The Third Hospital of Hebei Medical University, Shijiazhuang, P. R. China; 2 Department of Pharmacy, The Second Hospital of Hebei Medical University, Shijiazhuang, P. R. China; 3 Department of Orthopaedics, The First Hospital of Handan, Handan, P. R. China; 4 Chinese Academy of Engineering, Beijing, P. R. China; 5 NHC Key Laboratory of Intelligent Orthopeadic Equipment (The Third Hospital of Hebei Medical University), Shijiazhuang, P. R. China; Chongqing University Three Gorges Hospital, CHINA

## Abstract

**Objective:**

Acute compartment syndrome (ACS) is a serious medical condition that can be encountered in tibial plateau fractures. However, no studies of compartment pressure changes in patients with tibial plateau fractures compared to patient without fractures have been reported. To obtain a comprehensive understanding of the pressure changes in patients with fractures, we monitored and recorded the compartment pressure and attempted to reveal the potential pressure release function of the human fascia.

**Materials and methods:**

Cohorts of 43 normal individuals and 23 patients (initial 33, 10 were excluded due to inclusion criteria) and include the number of patients who completed the study with closed tibial fractures (the fracture group, FG, which comprised 6 men and 17 women) were included in this retrospective research. Compartment pressures were measured with Icare, a device that is traditionally used to measure intraocular pressure. Results of measurements at 6 different locations in the lower limb were recorded and compared for three days (days 2, 3, and 4 post fracture) between normal cohort (CG) and fracture cohort (FG) patients.

**Results:**

The compartment pressures were comparable at each pressure measurement site (upper, middle and lower) in patients of the CG and the FG. Compared with the CG patients, there was a significant increase in compartment pressure at the upper lateral location in 18-45-year-old patients in the FG (*P* = 0.013) and at the upper lateral (*P* = 0.004) and medial locations (*P* = 0.005) in 46-69-year-old patients, and the values tended to normalize over time. Compared with the contralateral normal limb of patients in the FG, there was a significant increase in compartment pressure at the upper lateral location in 18-45-year-old patients (*P* = 0.009) and at the upper lateral (*P* = 0.015) and medial locations (*P* = 0.016) in 46-69-year-old patients on the fractured side. Based on different fracture classifications, there were no significant differences in compartment pressure at the medial (upper, middle and lower) locations when compared with pressures at the corresponding lateral sites of measurement.

**Conclusion:**

The results of this study revealed that the fascial compartment as a whole can release the increased intracompartment pressure after fracture to prevent complications such as acute compartment syndrome caused by a continued increase in pressure. The Icare as a portable device, is potentially useful in compartmental pressure measurement especially in emergency room.

## Introduction

Acute compartment syndrome (ACS) is a serious medical emergency that compromises muscle capillary perfusion due to elevated pressure in the closed muscle compartment and is typically induced by a traumatic event, excessive physical training, military antishock trousers, medical history of anticoagulants, bleeding disorders, vascular obstruction, or vascular surgery [1,2]. It is considered an orthopedic emergency that is commonly encountered in high-energy fractures (15%) [3,4]. Decompression of the myofascial compartment is still considered the only treatment option for ACS. Failure or delay in performing a fasciotomy can lead to contracture, permanent motor and sensory deficits, infection, nonunion of fractures, the need for muscle excision, and, in severe cases, amputation and death. However, in our previous research, it was noticed that when blistering occurred in severe tibial plateau fractures, alleviation of compartment symptoms such as pain and weak pulse was monitored in the clinic, and then the risk of ACS occurrence was decreased [4,5].

Currently, the invasive measurement of intracompartmental pressure remains the primary objective, quantitative method of diagnosing ACS [6,7]. Although invasive techniques are useful for measuring actual ICP (intra compartment pressure), serious shortcomings exist, such as iatrogenic injury, pain, infection, and even falsely elevated ICP of the affected extremity ^2^. Furthermore, repetitive measurement or continuous monitoring of ICP is more dangerous and impossible with these invasive techniques although timely diagnosis is essential in cases of suspected CS to avoid delay in its diagnosis and treatment. Until now, a number of noninvasive technologies proposed as adjuncts to traditional measures of ICP or as replacements for ICP measurements have been examined experimentally and clinically [8]. Most of these technologies are noninvasive and can be classified into two major categories: surrogates of ICP based on alterations in mechanical properties (e.g., hardness of tissue, myofascial displacement) and tissue perfusion (tissue oxygen saturation) [9,10]. Icare is a handheld device that delivers a brief mechanical impulse and extracts soft tissue biomechanical parameters from the subsequent damped natural tissue oscillation curves. It was reported that the device can provide a reliable assessment of skin or muscle stiffness and demonstrate high reproducibility, particularly in the overall stiffness parameter [11]. Therefore, it can be used to obtain additional data for evaluation of compartment pressure.

There are many studies in which doubt regarding the feasibility of pressure measurement in fractured patients has been expressed [12]. Kelvin reported that increased compartment pressures are frequently seen in patients with tibial shaft fractures without ACS [13]. However, no experiments on trends in compartment pressure changes have been reported that allow a comparison between patients with tibial plateau fractures and normal individuals. To compare the pressure changes in tibial plateau fractures, this research involved retrospectively reviewing patients with tibial plateau fractures and recording the compartment pressure changes using Icare in different locations in normal individuals (normal control), fracture-bearing and contralateral limb (contralateral controls) of fractured ones.

## Materials and methods

### Patients and controls

The electronic medical records of patients with tibial plateau fractures (Schatzker’s classification) treated in our hospital with open internal fixation between January 2021 and December 2022 were extracted for our research. The retrospective study was approved by the institutional review board in our hospital and was performed in accordance with the ethical standards in the 1964 Declaration of Helsinki. The patients diagnosed with tibial fracture with or without ACS were identified from electronic medical records, and written informed consent was obtained from all enrolled subjects. All data were fully anonymized before we accessed them, and the IRB or ethics committee demand the requirement for informed consent. The clinical trial number of the research was NCT04529330. The patients were divided according to the World Health Organization (WHO) classification: young people (age <45 years); middle age (age 46–69 years); elderly (age 70 and above). The data were accessed for research purposes in 1/6/2023.

The inclusion criteria specified closed tibial plateau fracture patients who were older than eighteen years. The exclusion criteria were restrictive against patients with pathologic, extra-articular proximal tibial fractures and patients who were eighteen years or younger. Patients treated with other implants and those who underwent conservative management were also excluded. Patients with blisters that appeared after the blood draw on the second day of hospitalization as well as polytrauma patients were also excluded from the analysis. Radiographs were used to assess the fracture pattern, which was classified in accordance with the OTA and Schatzker classification. The normal visit patients in our clinic were recruited as normal controls, and tibial plateau fracture patients that need hospitalization and operation was recruited as fracture controls.

### Pressure measurement

Compartment pressures were measured with Icare (TA01, Icare Oy, Vanda, Finland) by one Author in the team. Icare is a handheld device that applies a fixed, brief mechanical impulse and calculates biomechanical properties based on the tissue’s inherent response. The participants were allowed to rest comfortably for approximately 5 min to get relaxed. The injured or normal leg was positioned flat on the patients’ bed, and the knee was extended to minimize variations in compartment pressure monitoring due to different positions. The device has a disposable probe that is propelled from the tonometer toward the skin with a speed of 0.25–0.30 meters/second by an electrical-pulse generator, which can create a magnetic field. The higher the compartment pressure, the shorter the length of time during which the probe touches the skin and the quicker it rebounds back into the tonometer. The speed of the probe as it moves back into the device causes a change in the magnetic field, which is captured by the tonometer, subjected to calculation, and converted into a pressure value (mmHg). Demographic data were recorded at the beginning of the test. Radiographs of the fractured leg were reviewed prior to the start of the procedure to identify the fracture site.

### Measurement position

**Fig 1:**
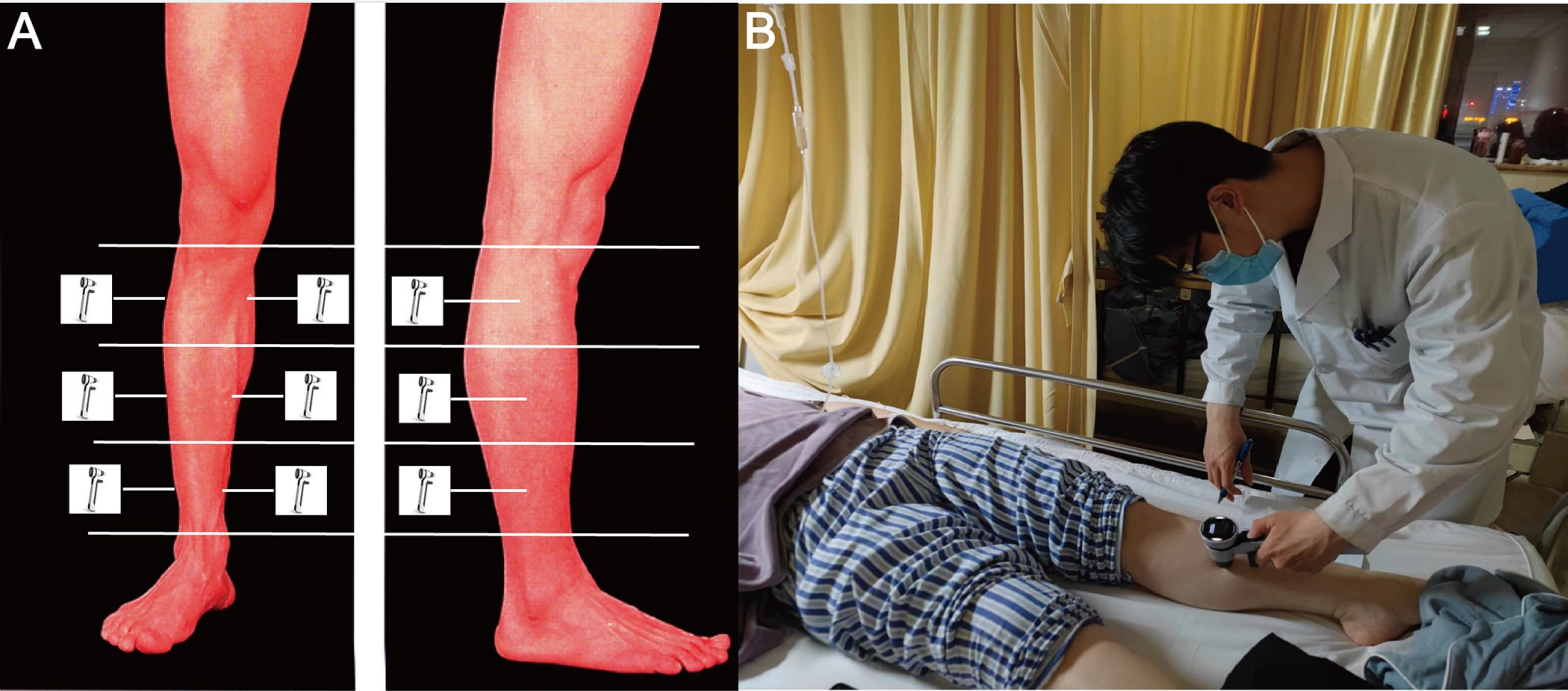
The measurement location of lower limb compartment pressure. A: The lower limb was divided into three identical parts, and the sites (upper, middle and lower points) for the anterior compartment were marked internally and laterally. B: Assessment points were marked and then used as measurement sites by orthopedic surgeons in normal and fractured patients.

The skin over the anterior and posterior superficial compartment of the lower leg was exposed for evaluation. The lower limb was visually segmented into three parts, and the sites (upper, middle and lower points) for the anterior compartment were marked internally and laterally. Assessment points were marked with a pen so that identical points were used for repeated measurements with the Icare device. Then, the compartment pressure was measured, results at 6 data points were recorded in each lower limb, and the average value was selected as the final result. Six measurements with 5-second intervals were guaranteed to minimize any potential measurement variation. Finally, the upper lateral and medial, middle lateral and medial, and lower lateral and medial compartment pressures were recorded in normal subjects for three days. Similarly, 6 measurements of the injured limb and contralateral normal limb were also obtained from tibial plateau fracture patients, the intra-observer reliability was calculated (**Fig 1**). Pressure measurement in different locations, 1, 2- and 3-days post fracture in two groups, comparison between injured and normal limbs in the FG group and the pressure based on different fracture classifications was respectively calculated and discussed. The normal subject do not need to revisit the hospital, if they live far away, the examiner will go to their community. Therefore, the additional revisit or clinical registration was not necessary.

### Statistical analysis

Continuous data are presented as the means with standard deviation. Homogeneity of variance for continuous variables was evaluated with the Levene test for equality of variances. Mann–Whitney–U tests were conducted for comparisons between two independent groups, and one-way ANOVA and LST tests were used to conduct comparisons among three independent groups. For all analyses in this research, significance was set at the P < 0.05 level. Before data analysis, statistical significance was set at P = 0.05. All analyses were conducted using SPSS Version 22.0 (IBM Corp, Armonk, NY). The intraobserver reliability was statistically analyzed by weighted kappa coefficients (SPSS 21, IBM, Armonk, NY, USA). According to the general requirements of statistics, taking α = 0.05, β = 0.1, the data were substituted into PASS11 software, and concluded that the minimum number of cases to be completed in each group is 12 cases.

## Results

33 patients with tibial plateau fractures were initially enrolled in this research; among them, 10 patients were lost to follow-up. Finally, 23 closed tibial fracture patients (named the fracture group, FG, and comprising 6 men and 17 women) were included in this research. Among the 23 tibial plateau fracture patients, 8 factures were due to falls from height, 7 were due to traffic accidents, 4 were due to falls from standing roads, and 4 were due to crushing injuries. There were 3 fractures identified as Schatzker Ⅰ, 7 as Schatzker Ⅱ, 3 as Schatzker Ⅳ, 6 as Schatzker V, and 4 as Schatzker VI. All of the patients were treated with a locking plate. Furthermore, 43 normal subjects) without lower limb injury were also enrolled in this research and comprised the control group (CG). There were no significant differences about sex, age and associated diseases (**Table 1**).

**Table 1:** Comparison of demographic data in CG and FG group.

	CG	FG	*P* value
**Sex**			
Male	13	6	
Female	30	17	0.723
**Age**	47.2±14.0	44.3±18.8	0.157
**Associated disease**			
Diabetes	3	2	
High blood pressure	9	3	
Normal	31	18	0.723

### Pressure measurement based on different locations

**Fig 2:**
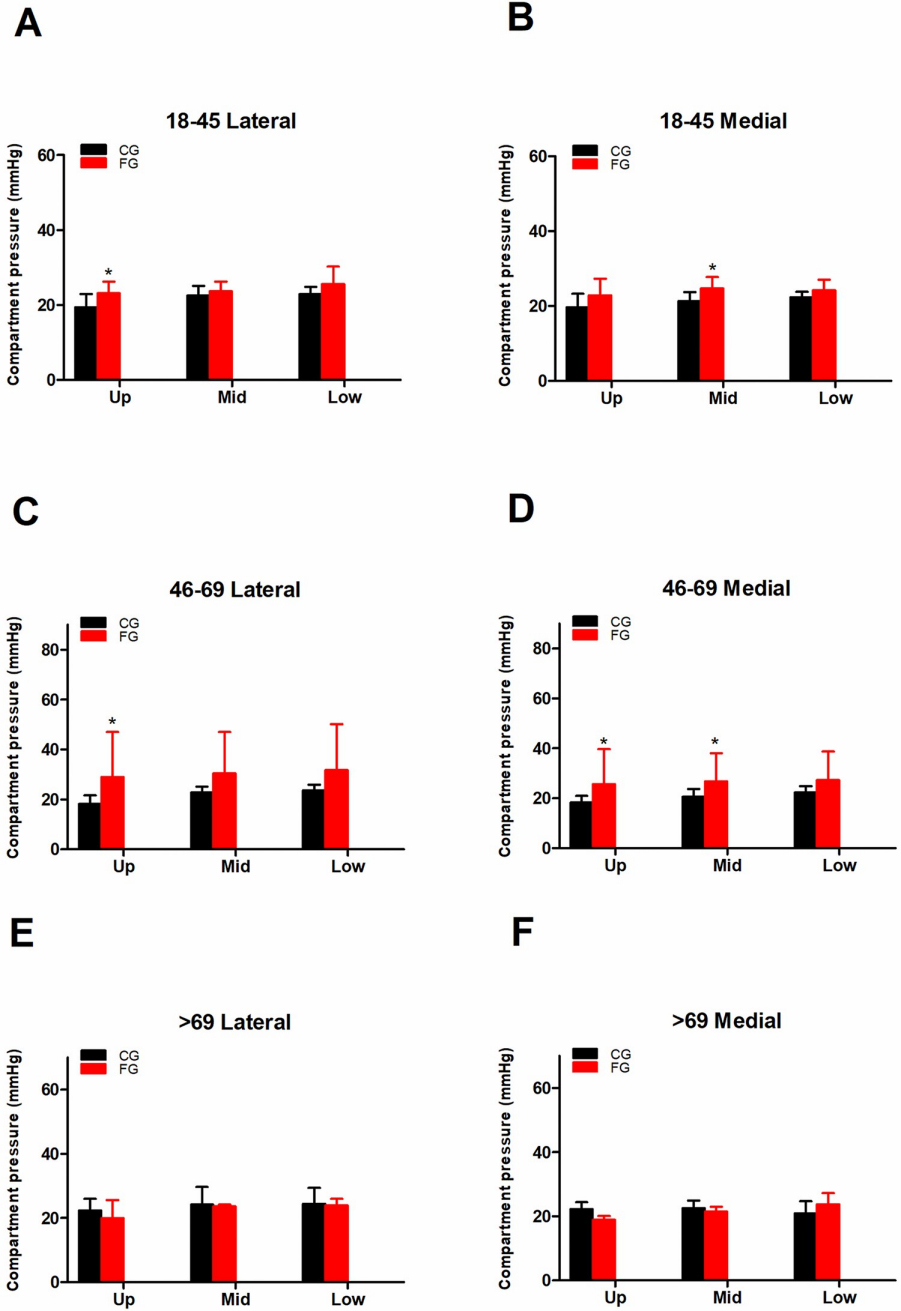
The pressure measurement result comparison between the CG and the FG on the second day post fracture based on different locations. A, B: Pressure results on the second day post fracture in the 18–45 age group. C, D: The pressure results on the second day post fracture in the 46–69 age group. E, F: The pressure results on the second day post fracture in the 18–45 age group.

The mean k value for the intra-observer reliability was 0.812, which indicated satisfactory agreement. There was a significant difference in the compartment pressure in the upper lateral and middle medial locations in the 18–45 age group of the CG and the FG. A significant difference was observed in the upper lateral, upper medial and middle medial locations in the 46–69 age group. However, no significant differences were observed in the >69 age group (**Fig 2**). The compartment pressures were comparable in each pressure measurement site (upper, middle and lower) of the CG and FG groups.

### The pressure comparison for three days in the CG and FG groups

**Fig 3:**
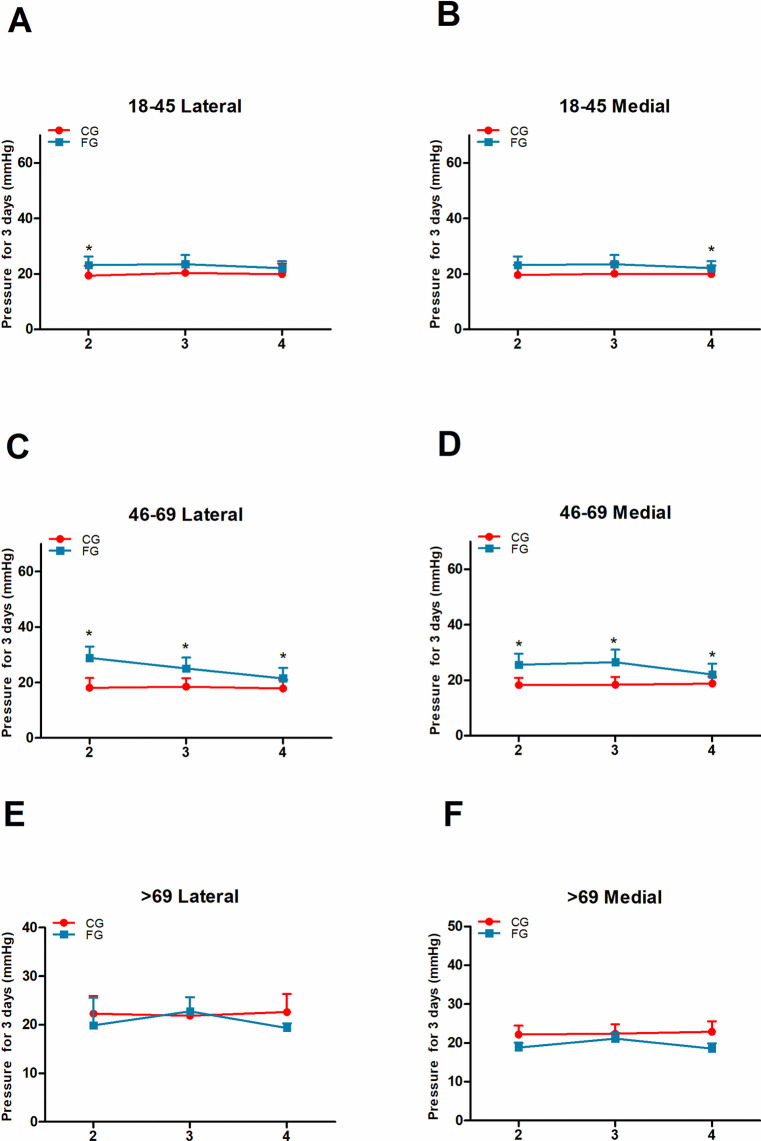
Pressure measurement result comparison between the CG and the FG three days post fracture at the upper lateral and medial measurement locations. A, B: Pressure results on the second day post fracture in the 18–45 age group. C, D: The pressure results on the second day post fracture in the 46–69 age group. E, F: The pressure results on the second day post fracture in the 18–45 age group.

On the second day after fracture, there was a significant increase in compartment pressure in the upper lateral location in 18-45-year-old patients in the FG (23.19 ±3.09 VS 19.4 ±3.51 mmHg; *P* = 0.013) and in the upper lateral (28.90 ±18.10 VS 18.1 ±3.52 mmHg; *P* = 0.004) and medial locations (25.60 ±14.03 VS 18.3 ±2.63 mmHg; *P* = 0.005) in 46-69-year-old patients compared with patients in the CG. For patients in the >69 age group, no significant differences were observed in the FG compared to the CG. On the third day, a significant increase in compartment pressure was observed only in the upper lateral (25.08 ±13.91 VS 18.5 ±3.05 mmHg; *P* = 0.009) and medial locations (26.53 ±14.46 VS 18.4 ±3.83 mmHg; *P* = 0.002) in 46-69-year-old patients in the FG compared with the CG. On the fourth day, a significant increase was observed in the upper medial location in patients aged 18–45 years (22.33 ±1.58 VS 19.9 ±3.03 mmHg; *P* = 0.017) and in the upper lateral (21.49 ±3.83 VS 17.9 ±3.06 mmHg; *P* = 0.007) and medial locations (22.10 ±3.92 VS 18.8 ±2.48 mmHg; *P* = 0.016) in patients aged 46–69 years in the FG compared with the CG. Based on the figures presented, the increased compartment pressure in the FG group was obvious (**Fig 3**).

### Pressure comparison between injured and normal limbs in the FG group

**Fig 4:**
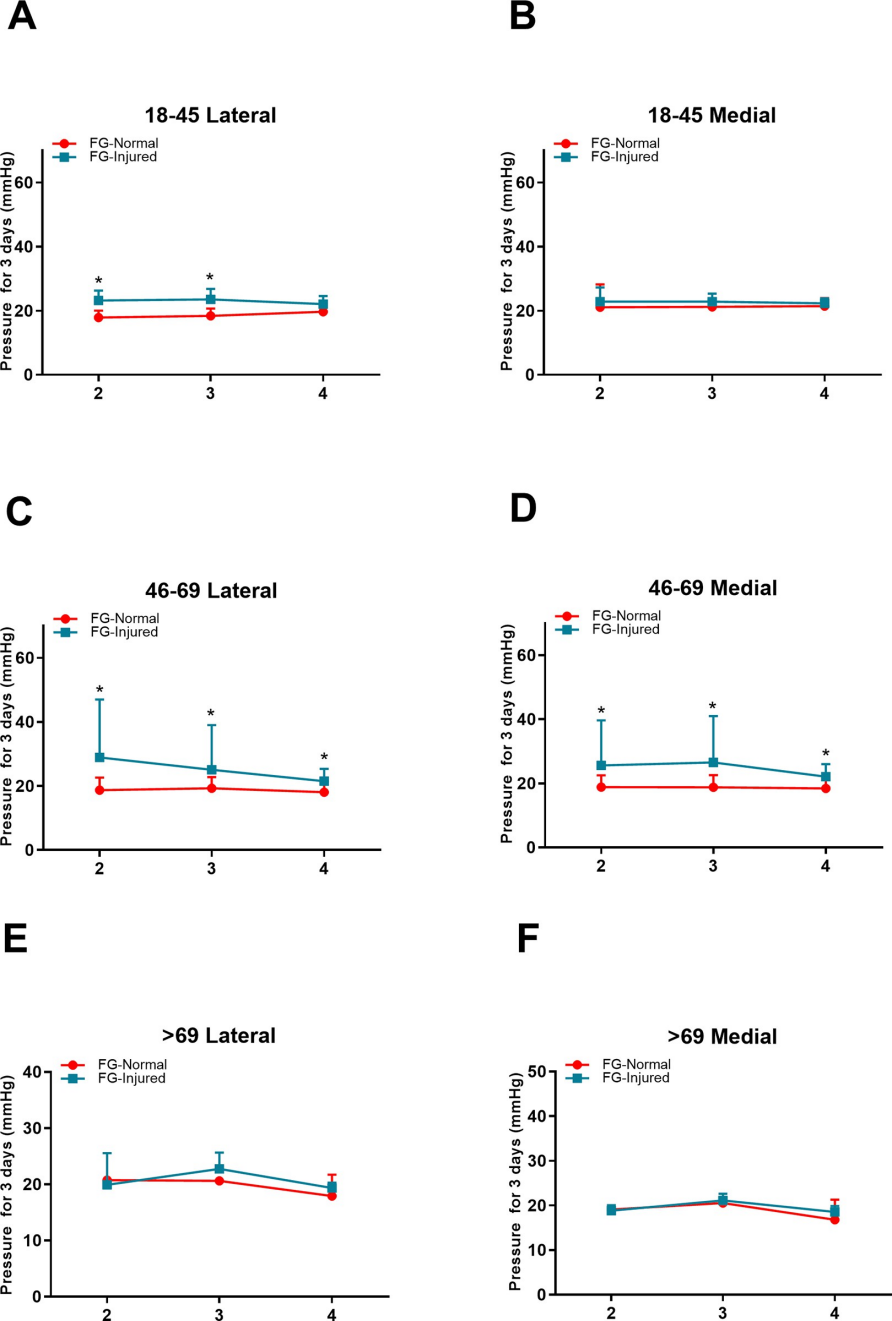
Pressure measurement result comparison between the normal and fractured sides in the FG three days post fracture at the upper lateral and medial measurement locations. A, B: Pressure results on the second day post fracture in the 18–45 age group. C, D: The pressure results on the second day post fracture in the 46–69 age group. E, F; The pressure results on the second day post fracture in the 18–45 age group.

On the second day after fracture, there was a significant increase in compartment pressure in the upper lateral location in 18-45-year-old patients in the FG (23.19 ±3.09 VS 17.86 ±2.20 mmHg; *P* = 0.009) and in the upper lateral (28.90 ±18.10 VS 18.68 ±3.90 mmHg; *P* = 0.015) and medial locations (25.60 ±14.03 VS 18.81 ±3.70 mmHg; *P* = 0.016) in 46-69-year-old patients compared with that of the contralateral normal limb. For subjects in the >69 age group, no significant differences were observed between injured and normal limbs in the FG. On the third day, a significant increase was also observed in the upper lateral location in 18-45-year-old patients in the FG (23.54 ±3.28 VS 18.4 ±2.29 mmHg; *P* = 0.003) and in the upper lateral (25.08 ±13.91 VS 19.28 ±3.51 mmHg; *P* = 0.022) and medial locations (26.53 ±14.46 VS 18.75 ±3.79 mmHg; *P* = 0.008) in 46-69-year-old patients compared with the CG. On the fourth day, a significant increase was observed in the upper lateral (21.49 ±3.83 VS 18.03 ±3.49 mmHg; *P* = 0.029) and medial locations (22.1 ±3.92 VS 18.42 ±2.82 mmHg; *P* = 0.02) in 46-69-year-old patients in the FG compared with the contralateral normal limb (**Fig 4**) **(S1 Data)**.

### The pressure comparison in different fracture classifications

**Fig 5:**
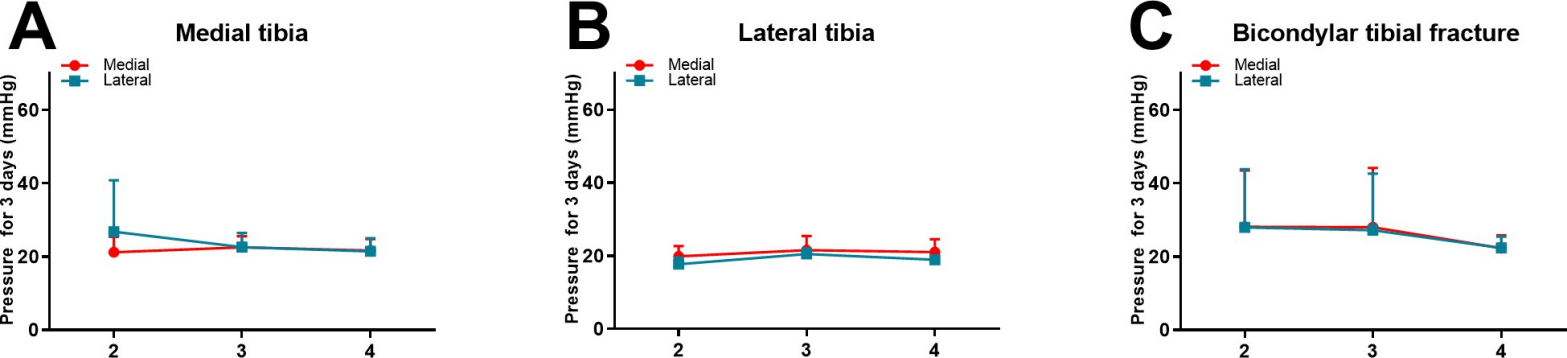
The pressure comparison in internal, external and bilateral tibial plateau fracture. A: Pressure results at the same location with monitoring over three days in medial tibial plateau fractures. B: Pressure results at the same location with monitoring over three days in lateral tibial plateau fractures. C: Pressure results at the same location with monitoring over three days in bicondylar tibial plateau fractures.

Based on different fracture classifications (medial, lateral and bicondylar tibial plateau fractures), it was observed that there were no significant differences in the compartment pressure in the medial (upper, middle and lower) locations when compared with pressures in the corresponding lateral locations. Furthermore, no significant differences in compartmental pressure according to between internal, external and bilateral tibial plateau fracture were found at the same location with monitoring over three days (**Fig 5**).

## Discussion

Acute compartment syndrome has always been one of the difficult problems encountered in the treatment of orthopedic fractures. In this study, Icare, a widely used instrument for measuring intraocular pressure, was innovatively used to measure the compartment pressure of the lower limb. The results showed that the compartment pressure in patients with tibial plateau fracture was significantly higher than that in normal-group patients; however, the pressure tended to decrease and return to normal with time. Therefore, no tibial plateau fracture patients in this research were found to have acute compartment syndrome or other intraoperative complications caused by the increase in compartment pressure. Compared to the pressure related ultrasound, which are widely investigated as a non-invasive measurement of muscle compartment elasticity, Icare was convenient and had a great potential for clinical application especially in the emergency room [14–21]. Furthermore, the study reveals that the fascia compartment is a whole structure that can release highly increased pressure through a particular potential mechanism such as the opening of paracellular pathways by PI3K/Akt/Claudins [22].

The results of previous studies have suggested that when the pressure in the fascia increases to a certain value (diastolic pressure minus compartment pressure <30 mmHg), the risk of acute compartment syndrome is significantly increased. However, high compartment pressures do not equate to the occurrence of acute compartment syndrome, and relative excessive treatment measures such as fasciotomy should therefore be avoided [5,13]. Furthermore, there have been no reports of research involving an attempt to monitor compartment pressure changes with this facilitated device in the clinic. In our research, the Icare device was innovatively used to measure the changes in compartment pressure, and the compartment pressure of patients with tibial plateau fracture and normal controls was measured continuously for 3 days. It was found that the pressure in fractured patients exhibited a downward trend with time rather than remaining the same. Two reasons can be proposed to explain the results. The first reason is that with the reduction in pain and swelling, the muscle tension of the fractured lower limb decreases slowly, so the compartment pressure decreases. Second, the fascial compartment is a continuous cavity structure, so the pressure is dispersed and released by pressure redistribution. Consistent with this hypothesis, the experimental results of this study revealed that the pressure values at six pressure measurement locations on the same day were comparable and were not significantly different between patients in the CG and the FG; this confirms that the fascia cavity is an integrated space that allows for pressure dispersion.

In addition, compared with the CG, it was found that the closer to the fracture site, the greater the pressure in the fascial compartment. It was also observed that the differences in pressure in the upper lateral and middle medial location of patients in the 18–45 age group as well as differences in pressure at upper lateral, upper medial and middle medial locations in patients of 45–69 years of age were statistically significant; however, no significant differences were observed in patients over 69 years old. The reason for this observation regarding patients over 69 years of age may be that there are fewer people in this age group. In addition, the muscle tissue of elderly patients is reduced, and the fascia and surrounding skin are more relaxed, so the increased compartment pressure can be released quickly. In addition, the research results show that there is no statistical significance in the pressure difference between the upper medial and lateral locations based on different fracture classifications. The reason for this may be that the pressure in the fascia is determined not only by the fracture type but also by the patient’s age. However, the trend of greater compartment pressure with increasing proximity to the fracture site is still obvious. Furthermore, the results of some studies suggest that the best location for measurement of compartment pressure is 5 cm within the fracture site. However, the results of this study do not support this point completely, probably because the compartment cavity is an intact cavity, and the measured value would be expected to the same or similar even if the measurement site is far away from the fracture site. The finding of comparable measurement results in the middle and lower locations compared with those of the upper locations strongly support our hypothesis; this poses a direct question regarding the previous assessment of best positioning for pressure measurement.

To further verify the accuracy of the research results, the compartment pressure on the fracture patient’s contralateral side was also measured and compared with the pressure associated with the fracture. The results showed that the compartment pressure at the upper lateral location in patients of the 18–45 age group and the upper lateral and medial locations in patients of the 46–69 age group was significantly higher on the side with fracture than on the healthy side; however, this difference only persisted in the age group of 46–69 years, the difference continuing to be observed until the fourth day. The reason is that patients in the age group of 18–45 are younger; given a faster metabolic rate in these patients, the fascia tissue itself and the skin and other structures contribute to releasing the pressure in the compartment more quickly. Our previous research also confirmed that the increase in pressure in the fascia compartment can promote opening of the paracellular pathway of skin and accelerate the formation of blister fluid. Furthermore, the formation of blisters has also been confirmed as a manifestation of pressure release [4,22]. The reason for our choosing only the upper two measurement points is that the measurement results at the middle and lower parts of the measurement point were similar to those at the proximal measurement location, so the measurement results at the proximal location may better represent the trend of changes.

## Limitations

The patients enrolled had a relative high rate of lost follow up, which will have an effect on the results. In our following research, more rigid criteria for inclusion will be set, and more patients will be enrolled to minimize its impact on research results. Second, we have doubted the judgment about the best pressure measurement position but still have not put forward a new pressure measurement method and position, so further exploratory research is needed. Thirdly, patients with blisters after fracture were not included in the study because the number of people measured was too small for them to be added to this study for comparison. In our follow-up study, patients with tibial plateau fractures with blisters will be included to examine the changes in intracompartment pressure before and after blisters appear in our following research. Fourthly, the Icare which was widely used, need to be verified in animal model in our following research.

## Conclusions

Above all, the results of this study revealed that the fascial compartment as a whole can release the increased intracompartment pressure after fracture to prevent complications such as acute compartment syndrome caused by a continued increase in pressure. The Icare as a portable device, is potentially useful in compartmental pressure measurement especially in emergency room.

## Supporting information

S1 Data The relevant original data used in this research was presented in S1 Data. (XLSX)
